# Response to “Winner of the Ronald Melzack — *Canadian Journal of Pain 2021* Paper of the Year Award”

**DOI:** 10.1080/24740527.2022.2108775

**Published:** 2022-09-15

**Authors:** Jason W. Busse, David Juurlink, D. Norman Buckley

**Affiliations:** Department of Anesthesia, Michael G. DeGroote School of Medicine, McMaster University, Hamilton, ON, Canada; Department of Health Research Methods, Evidence and Impact, McMaster University, Hamilton, ON, Canada; Sunnybrook Health Sciences Centre, Toronto, ON, Canada; Departments of Medicine and Paediatrics, University of Toronto, Toronto, ON, Canada

The commentary of Dr. Katz and Ms. Waisman^1^ misrepresents the 2017 Canadian Guideline for opioid therapy and chronic noncancer pain. The authors claim: “The winning article documents the ongoing harms done to Canadians with chronic pain in the wake of the 2017 Canadian Guideline for Opioid Therapy and Chronic Non-Cancer Pain that introduced strict dose limits for people taking opioids, fostered a climate of fear among prescribers, and opened a Pandora’s Box of anguish and helplessness among patients” (p. 121). The commentary also fails to place the challenges faced by people living with pain in the context of strong public criticism of prescribing practices, actions by several Canadian provincial medical regulatory bodies, and guidelines arising from the United States, in particular the Centers for Disease Control and Prevention (CDC)^2^ and the U.S. Department of Veterans Affairs and Department of Defense,^3^ all stridently calling for reduced use of opioids for chronic pain. Temporally, the reduction in opioid prescribing predates (by several years) the release of the 2017 guideline.^4^

The referenced article^5^ interviewed 22 participants, all of whom were selected based on their report of having been stigmatized by the opioid epidemic. The authors identified five themes, including opioid tapering as a standardized response from providers; some participants reported being pressured by their physicians to taper against their wishes or tapered more quickly than they wished. The article concluded: “Opioid guidelines are essential to inform clinical practice, but they should be interpreted in the context of a comprehensive provider–patient interaction in which the patient’s preferences, feelings, and values are addressed” (p. 75).

This recommendation is exactly consistent with the 2017 Opioid Guideline, which concludes with the statement: “… tapering efforts should be individualized and should consider patients’ values and preferences” (p. E665).^6^

The relevant recommendation in the Canadian guideline (Recommendation #9) suggests tapering opioids to the lowest effective dose for patients with chronic noncancer pain who are using ≥90 mg morphine equivalents of opioids per day. This recommendation was a weak one based on low-quality evidence that suggested reducing opioid dose may decrease the risk of unintentional overdose and in consideration of risks associated with opioid tapering including withdrawal. A weak recommendation means that though most informed patients would choose the recommended course of action, an appreciable minority would not. With weak recommendations, clinicians should recognize that different choices will be appropriate for individual patients, and they should help patients arrive at decisions consistent with their values and preferences. The final decision to attempt a trial of opioid tapering rests with the patient. This recommendation is accompanied with the following remark: “Some patients are likely to have a substantial increase in pain or decrease in function that persists for more than one month after a small dose reduction; tapering may be paused and potentially abandoned in such patients” (p. E662).

The 2017 Guideline was particularly cognizant of the potential harms associated with inappropriate opioid tapering for two reasons:

(1) As we wrote in our 2016 commentary, a year before the Canadian guideline was published:

The CDC [guideline] provides a strong recommendation to avoid increasing the dosage of opioids to 90 morphine milligram equivalents (MMEs) or more per day for patients with chronic noncancer pain. In a major omission, the guideline fails to address clearly how clinicians should manage patients currently prescribed dosages that are in excess of 90 MME per day. … Overly aggressive adoption of the CDC guideline may lead to harm if physicians try to abruptly transition patients already receiving opioids at high doses to much lower doses. Harms could include withdrawal reactions, uncontrolled pain, anxiety for patients and loss of trust in their physicians. Such consequences could leave patients desperate. There is already preliminary evidence that in British Columbia, where the CDC guideline recommendations have been adopted as standards of practice, some patients have sought illicit opioids in the wake of reduced prescribing by physicians. With the profusion of counterfeit fentanyl in Western Canada, the consequences could be fatal (p. 1210).^7^

(2) Our exploration of patient values and preferences identified the following:

Our focus group interviews revealed that some patients using long term opioid therapy for chronic non-cancer pain were concerned about adverse consequences of opioid withdrawal that may result from efforts to wean or discontinue their opioid use. For those using high doses of opioids in whom weaning is undertaken, we continue to place a high value on societal considerations of minimizing the risk of rare serious adverse events, but we also place a high value on avoiding severe suffering, increased pain, and functional limitation that may accompany opioid reduction. We also place a high value on patient autonomy under these circumstances (p. 1).^8^

The 2017 Opioid Guideline did not introduce strict dose limits for people taking opioids. The Guideline did encourage clinicians to approach patients prescribed high-dose opioid therapy to consider a voluntary trial of tapering that, in patients who elected to proceed, could be paused or discontinued based on their response. A growing body of evidence indicates that voluntary, supported opioid tapering can help most people who live with chronic pain who are prescribed high-dose opioid therapy substantially reduce their dose without increased pain or decreased function.^9,10^
